# Supplementary material for: Are carbon emissions trading and green financial instruments synergistic? -Comprehensive quantitative research based on content analysis
Source: PLoS One. 2024 Mar 7;19(3):e0298601. doi: 10.1371/journal.pone.0298601 (PMC10919601; doi:10.1371/journal.pone.0298601)
Supplement: S1 Data — (ZIP) [file pone.0298601.s001.zip › Data/地级市Z_F工作报告.zip!/║■▒▒/╨ó╕╨╩╨/└┤╫╘╣1⁄2╓┌║┼í╛┬φ┐╦╩2╛▌═°í┐_2015.docx]

各位代表：

　　我代表市人民政府向大会报告工作，请予审议，并请市政协委员和列席人员提出意见。

　　一、2014年工作回顾

　　过去一年，在省委、省政府和市委的坚强领导下，在市人大、市政协的监督支持下，市政府团结和依靠全市人民，认真贯彻落实党的十八大、十八届三中、四中全会和习近平总书记系列重要讲话精神，以党的群众路线教育实践活动为契机，围绕大力推进“五个跨越”、加快建设“五个城市”〔1〕，坚持“竞进提质、升级增效”不动摇，积极适应新常态，保持定力谋发展，全市经济持续平稳运行，民生改善呈现新亮点，主要经济指标总量在全省处于4—6位，增幅高于全省和武汉城市圈平均水平，较好完成了市五届人大四次会议确定的目标任务。

　　——坚持稳增长与谋长远结合，实体经济有新发展。工业经济持续扩规。深入推进“三大工程”〔2〕，改进服务企业方式。新增规模以上工业企业182家、达到1148家；新增销售收入过10亿元企业5家、达到28家；新增过百亿元产业聚集区1个、达到6个。新增高新技术企业43家、达到125家。新增省级以上研发平台10个、达到56个；新增院士工作站19家、达到32家。新增中国驰名商标2件、湖北名牌产品41件。新增建筑业产值79.2亿元、达到390亿元。现代农业发展加快。坚持以工业化理念谋划农业,加速推进农业产业化。粮食生产实现“十一连增”。农业特色板块基地达到550万亩。农村土地流转率达到27.3%。农民专业合作社达到5788家，数量全省第一。实现农产品加工产值1200亿元，增长12%。完成农田水利建设投资20亿元，新解决25.5万人饮水安全问题。植树造林17.1万亩。新建通村公路1019公里。主要农作物机械化作业水平达到72.5%。开通了农业气象“直通车”服务。减少农村贫困人口3.86万人。完成第四轮“三万”活动。服务业发展势头向好。鼓励“小进限”，新增“限上”企业〔3〕145家、达到1025家。升级旅游休闲消费，接待游客1600万人次，旅游综合收入突破100亿元，分别增长14.3％和19.1％。扩大信息消费，4G信号覆盖率达到90%。电力、烟草、邮政、通信、石油、盐业等行业实现新发展。争取重大政策性项目成效显著。成功获批国家新型城镇化、信息惠民、养老服务、低碳工业园区、农产品流通体系建设等一批国家试点项目。滚动实施100个重大工程项目，完成年度投资225亿元；31个省级重点项目完成投资117亿元。武西铁路孝感段、安陆火电厂等重点项目前期工作进展顺利。

　　——坚持抓改革与促开放结合，市直“三区”、县域经济显现新活力。全面启动深化改革工作。全市共承担国家级改革试点项目22项、省级32项。对涉及市政府层面的重点改革项目，按照时间节点有序推进。完成食品药品、卫生计生、文化体育、新闻出版广电等机构改革。组建孝感日报传媒集团、广电传媒集团。“两型”社会专项改革试验取得新进展，大别山试验区生态保护得到加强。运用改革办法破解发展难题。引进汉口银行、民生银行，组建孝感农村商业银行，全市金融机构新增贷款117.3亿元。注重多层次融资，富邦科技成功上市，18家企业在“新三板”〔4〕、“四板”〔5〕挂牌。市城投公司、市高创投公司分别发行16亿元企业债券，争取棚户区改造政策性融资38亿元。整合9000万元财政资金，撬动11亿元贷款扶持实体经济发展。推进工商登记制度改革，市场主体突破28万户。开展节约集约用地专项整治，盘活存量土地10098亩。规划建设252个“三类”新社区〔6〕，开工174个，节约土地3450亩。实施“槐荫人才计划”，列支550万元专项资金用于人才的引进、培养和奖励。加强“三支队伍”〔7〕建设，打通服务群众“最后一公里”。大力推进回归创业工程，新创办投资500万元以上回归企业690家。深度推进对外开放。开展务实招商，引进省外资金735亿元，增长15.3%。突出产业链招商，孝南纸制品、汉川食品、应城化工、云梦皮草、安陆粮机、大悟风能、孝昌机械电子等产业新引进配套项目140个。黄麦岭磷化公司与省联发投实现战略合作。新批外商投资企业29家。新增出口实绩企业57家、达到230家。孝昌鸿翔鸭业成为全省第一家肉禽自主出口企业。市直“三区”跨越发展的基础不断夯实。市高新区20个产业项目竣工投产、62个开工建设、26个签约筹建。新引进落户世界500强和上市公司11家，日本小糸、中央发条、友成控股等一批日资企业扎堆发展。军民融合产业示范基地实现产值145亿元，增长11%。市临空经济区完成基础设施投资17亿元，“四纵六横”骨干路网加快形成，推动白水湖大桥武汉段正式开工,普洛斯、申通等物流项目动工建设。市双峰山旅游度假区核心景区详规提档升级，续建和新开工项目5个。推动县域经济竞相发展。孝南50%的乡镇财政收入过5000万元，区级财政总收入突破20亿元；汉川新增规模以上工业企业51家，总数占到全市的三分之一；应城实施技改扩能项目143个，传统盐化产业加快转型升级；云梦外贸出口逆势上扬，增幅全省第一；安陆“一区五园”〔8〕活力显现，园区工业增加值占该市的76.6%；大悟引进新能源项目7个,“华中风电之都”雏形初现;孝昌苗木花卉、生态旅游等产业快速发展，机械电子产业产值占该县的40%。

　　——坚持城市建设与环境治理结合，城乡面貌呈现新变化。以全域理念完善城乡规划。《孝感市城市总体规划》获省政府批准，6个县市城市总体规划完成修编。规划管理得到加强，市区一体控违格局初步形成。全市城镇化率提高到52%。以建设和改造并举提升中心城区品位。孝感城区实施城建项目110个，完成投资50亿元。主城区实施7条骨干道路改造工程，完成44条背街小巷基础设施改造提升工程；东城新区25个重点建设项目顺利推进；南城新区骨干路网建设进展顺利。以延伸两个“六张网”〔9〕为抓手完善城镇功能。6个县市城区实施城建项目145个，完成投资29.7亿元。城镇公共服务网络向农村延伸，覆盖面大幅提高。“四城同创”〔10〕、文明卫生村镇创建、新农村“一线一点”〔11〕建设扎实推进，9个镇入选新一轮全国重点镇，孝感市连续五届荣获省级文明城市称号。以市场取向推进城市管理创新。孝感城区实施环卫作业市场化、路段管理标准化、市政维护专业化“三项改革”，引入城市综合管理第三方考评机制，城市管理水平得到提升。以前瞻思维改善生态环境。完善重点湖泊、主要流域保护性开发长效机制，落实国控重点污染源企业在线监控制度，推进大气污染防治年度计划。投资9.3亿元实施减排项目113个，节能减排完成省定任务。在孝感、应城、云梦、大悟城区新建水厂4座。孝感和6个县市城区的污水处理厂全部建成。孝感城区新增绿地150万平方米，槐荫公园三期基本建成，6.5公里绿色生态走廊初步形成。

　　——坚持保基本与促公平结合，民生改善取得新成效。社会事业不断进步。教育公平进一步体现，5个县市区义务教育均衡发展通过国家认定、2个通过省级评估，市直“三区”教育教学条件明显改善；新改扩建农村标准化学校52所，招录1038名大学生到农村任教，全年资助贫困学生6.2万人。卫计工作有机融合，实施卫生基础设施项目27个，启动300名新型乡村医生培养计划；强化出生人口性别比综合治理，落实“单独二孩”政策。文化体育惠民工程取得新进展，开工建设市文化中心，改造完成市体育中心，建成30个村级文化广场。孝文化活动丰富多彩，成功举办第11届中华孝文化旅游节，承办重阳与亚洲孝道文化国际论坛。应城、安陆被评为全国文化先进县市。驻孝部队在承担急难险重任务、服务地方经济社会发展等方面，发挥了积极作用。国家安全、人民防空工作有新进展，老龄、妇女、儿童、残疾人事业健康发展，民族宗教、外事侨务、统计调查、对台、地方志、档案、保密、机关事务管理等工作取得新成绩。民生保障不断完善。明确政府部门主体责任，扎实推进党的群众路线教育实践活动中确定的100件为民实事和市政府承诺的十件实事。就业创业扶持政策有效落实，“双助”行动〔12〕力度加大，新增城镇就业6.3万人，救助困难群众34万人次。“五项社保”〔13〕实现增覆扩面，新增参保人数9.3万人。城镇职工和居民住院报销比例分别提高3个和1.5个百分点。“五项社会救助”〔14〕标准大幅提高，发放救助资金增加1.5亿元。新改扩建农村福利院21所，市、县两级社会福利中心实现全覆盖。开工建设保障性安居房4.4万套，建成1.96万套，改造农村危房4126户。住房公积金管理体制理顺。居民消费价格涨幅2%。社会治理不断创新。注重运用新方式、新手段，有效应对综治、信访、维稳、安全生产新形势。深化平安孝感建设，构建立体化社会治安防控体系，在全省率先推行“一村（社区）一警”，在市、县城区建成社会治安视频监控系统，在城乡实现网格化管理全覆盖。食品药品、产品质量、安全生产形势总体平稳，信访、社区矫正工作位居全省前列，社会保持和谐稳定。高质量完成第九届村民委员会换届选举工作。

　　——坚持改进作风与强化监督结合，政府自身建设得到新加强。作风转变成效明显。党的群众路线教育实践活动中，市政府党组广泛征求意见，明确了5个方面、35项整改任务。坚持不回避矛盾，积极回应群众关切，21项年度整改任务已经完成，14项需要长期整改的任务按时间节点有序推进。落实“三短一简”〔15〕要求，倡导“一线工作法”，采取部门联动、现场办公的方式，推动工作在一线落实。依法行政能力提升。市政府建立完善六大决策委员会〔16〕，对国土、规划、财政资金、国有资产、投融资等重大敏感事项，实行集体议事决策。主动接受人大及其常委会和政协的监督，市政府班子成员领办2—4件建议、提案，共承办市人大代表建议96件、市政协委员提案244件。全面清理市政府现行规范性文件，公布市级行政权力清单〔17〕，行政许可事项由310项减少到90项。深化行政复议相对集中改革，办理行政复议案件158件。党风廉政建设主体责任较好落实。坚持“一把手”负总责，班子成员“一岗双责”，切实履职履责，加强督导检查，强化执纪监督，市政府班子每季度开展一次警示教育活动，全市政府系统党风廉政建设取得实效。加大行政监察和审计监督力度，推动公共资源交易体制改革，对工程建设领域、财政性资金和民生项目实行全过程监管。完善民生工程项目公开征集、公示、听证、评议等办法，探索同类项目整体打包招标建设，推行工程质量终身负责制，从源头上预防腐败。

　　各位代表！过去的一年，我们在困难中奋力前行，在竞争中创新突破，实现了好于预期的经济社会发展。这是省委、省政府和市委坚强领导的结果，是市人大、市政协大力支持的结果，是全市人民艰苦奋斗的结果。在此，我代表市人民政府，向全市人民，向各位人大代表、政协委员，向离退休老同志，向各民主党派、工商联和社会各界人士，向驻孝部队、武警官兵、公安干警，向所有关心支持孝感改革发展的同志们、朋友们，表示衷心的感谢和崇高的敬意!

　　在总结成绩的同时，必须清醒地看到，政府工作仍然存在一些困难和问题。主要是：在宏观经济下行的大背景下，支撑发展的大项目、大产业还不够多，经济转型升级、保基本运转、保民生支出的压力较大；在资源环境约束偏紧的情况下，面临着保持合理的发展速度与保护好生态环境的双重压力；在人民群众多样化的需求下，民生改善的力度、城乡公共服务的均等化离人民群众的期盼还有不小的差距；在全面推进依法治国的新形势下，运用法治思维和法治方式、遵循市场法则解决问题的能力还需进一步提升，改进作风、推进清廉政府建设任重道远。对这些问题，我们将通过改革创新，采取综合措施切实加以解决。

　　二、2015年工作安排

　　2015年，政府工作的总体要求是：坚持以中国特色社会主义理论体系为指导，深入贯彻落实党的十八大、十八届三中、四中全会和习近平总书记系列重要讲话精神，贯彻落实中央、省委省政府和市委重大决策部署，围绕加快建设武汉城市圈副中心城市、打造“湖北的苏州”和建设“魅力孝感”战略目标，坚持竞进提质、升级增效，主动适应新常态，有效引领新常态，全面推进深化改革和法治孝感建设，持续推进民生改善，深入推进作风转变，奋力完成“十二五”目标任务，谱写大力推进“五个跨越”、加快建设“五个城市”和孝感第三次创业新篇章。

　　（一）把做大做实有质量的经济总量作为政府工作的首要任务。继续做大做强工业，发展有效益的农业，提升第三产业比重，促进一二三产业融合互动发展。

　　处理好扩规模与调结构的关系，提升工业经济质效。研究深化推进“三大工程”的新途径、新办法，抓龙头、补链条、壮产业、创品牌、强管理、拓市场。加快推进100家骨干企业技改扩能、兼并重组，新增销售收入过10亿元企业3家。选择1000家成长性好的中小企业，开展“把脉问诊”，推动优化升级，新增规模以上企业100家。做实小微企业孵化平台，鼓励支持个体转企业，新增小微企业1000家。出台促进24个优势特色产业聚集区发展的政策措施，设立院士工作站，鼓励引导产业链项目聚集发展，新增过百亿产业聚集区2个。立足调优结构，加快推进新材料、高端装备制造、光电子信息、生物医药、新能源等战略性新兴产业发展。促进建筑业健康发展。以国家信息惠民城市试点为契机，推进“两化”融合〔18〕，让信息技术服务市场主体、方便人民群众。创新政府服务企业方式，“一对一”、“实打实”解决企业发展难题。

　　以“三农三化”为目标，加快发展现代农业。建基地、抓加工、创品牌、增效益，推动农业产业化提档升级。重点完善3个省级、4个县级农产品加工园区功能，叫响地方特产品牌，提高农产品效益，农产品加工产值增长12%以上。着力发展以种养大户、农民合作社、家庭农场为主的土地适度规模经营，农村土地流转率提高到30%以上。以提升农民职业化水平为目标，培训新型职业农民3300人次。实施“美丽孝感、清洁乡村”工程，推动“一线一点”新农村建设和新一轮“三万”活动取得新成效。加强农田水利、农业装备、农村电网升级改造建设，打通农技、气象服务“最后一公里”，粮食总产稳定在45亿斤左右。实施精准扶贫，减少贫困人口3.5万人。

　　坚持改造提升传统服务业和培育发展新兴服务业态，提升第三产业比重。制定出台支持服务业加快发展的具体措施，重点培育一批骨干商贸企业，引进一批大型商贸企业，扶持本土商贸企业做大做强。新增“限上”企业80家。加快物流园区建设，完善物流通道网络，推进生产、仓储、交易物流中转无缝对接。加快发展电子商务、快递服务、文化创意、健康养老等新兴服务业，引导和扩大个性化、多样化消费。完善农村消费平台，健全供销社网络体系，支持电子商务向农村延伸，方便农民群众就近消费。创新金融服务方式，加大支持地方实体经济发展力度，新增贷款126亿元以上。加强多层次资本市场建设，力争14家企业在“新三板”、“四板”挂牌。提升孝感旅游品牌，重点发展一日游和二日游，旅游综合收入增长15.9%。做强皮草、化工、机电三大主导出口产业，抓好出口行业、出口产品的多样化，帮助更多的企业实现自营出口，新增出口实绩企业30家。

　　做到争取、引进、建设同步推进，把项目作为做实经济总量的重要支撑。加大项目的谋划力度，结合编制“十三五”规划，研究国家重大产业政策，加强对长江经济带、汉江生态经济带、汉孝大临空等战略规划的研究,加强与鄂北水资源配置工程、武西铁路、天河机场三期等重点工程的对接，力争更多的项目纳入国家、省相关战略规划。研究新常态下招商引进的新办法，继续坚持“三抓三不放”〔19〕，围绕产业结构优化、产业链条延伸、产业集群发展，开展有针对性、专业性、实效性的定向招商。对已经引进落户、产能发挥不够充分的项目，逐一研究，帮助盘活存量或开展二次招商。力争新引进投资过亿元项目65个，其中过10亿元的10个。细化100个重大项目的年度建设清单，按时间节点强力推进。打击“四霸六强”〔20〕，优化项目建设环境。

　　（二）把推进新型城镇化作为统筹区域发展的重要抓手。最大限度用足用活国家新型城镇化试点城市政策，坚持全域孝感理念，一张蓝图绘到底，从严执行和管控规划，统筹区域发展。城镇化率提高到52.6%。

　　配套提升孝感城区整体功能，加快推进“六大组团”〔21〕建设。继续加强主城区背街小巷综合治理，加大棚户区改造力度，改善城市出入口环境；开展孝感城区地下管网普查。加快推进东城新区重点项目建设，改造提升董永路，完成乾坤大道延伸、澴川路扩建工程；加快配套城市功能，吸引人口聚集。着力抓好临空区水、电、路、气、污水处理、城市防洪排涝等市政基础设施建设。突出孝文化和生态两大元素，加大项目招商和建设力度，推动生态文化旅游区取得实质性进展。实施天仙南路、北京南路拉通工程，打通南城新区与主城区路网对接；加快推进老澴河综合治理工程。支持云梦实施城区东进战略，加快云孝交通对接。推进孝汉交通一体化，力争孝汉大道三期全线贯通、汉孝城际铁路建成通车。推进316国道改扩建，开工建设107国道外迁工程，启动孝感至汉川、孝感至应城一级公路建设。推进“四城同创”，深化城市综合管理考评机制。实施城市畅通工程，组织“城管综合会战”，重点解决孝感城区交通拥堵、占道经营、噪音污染、广告乱设、站前广场混乱等市民反映强烈的问题。

　　坚持城市新区、产业新城“双轮驱动”，统筹推进县市城区、小城镇和中心村建设。支持汉川城北、应城城南、安陆河西、大悟高铁、孝昌澴西等城市新区建设，通过产业带动就业、聚集人口。以“四化同步”〔22〕试点镇为示范，以30个市级重点镇为依托，打造一批具有浓郁本土特色的工业强镇、农业大镇、商贸重镇、文化名镇、旅游旺镇。以建设新社区为载体，通过镇村联动、有序集并、整体搬迁等措施，建设新农村。加强特色古镇、传统村落保护。推进户籍制度改革，实行居住登记制度，加快人口市民化进程。

　　以壮大产业为核心，强力推进市直“三区”跨越式发展。支持市高新区围绕五大产业聚集区进行招商，做强军民融合示范基地，实现产业聚集发展；加快建设8个新社区，落实失地农民保障机制；学习借鉴上海自贸区经验，创新服务、孵化、融资平台，引领“一区四园”发展。新签约、开工、竣工项目各20个，新增规模以上工业企业15家。支持市临空经济区抢抓汉孝大临空建设机遇，加快与武汉对接步伐；加大招商和落地项目建设力度，力争普洛斯项目建成运营；完成基础设施投入20亿元以上，力争3个新社区全面竣工。支持市双峰山旅游度假区推动核心景区标准化建设，加强生态保护，提升景区品位，加快打造成为华中地区旅游品牌、武汉城市圈重点旅游目的地。

　　发挥各地特色优势，协调推进县域经济争先进位。加大重大项目、产业发展、公共资源配置、政策资金的统筹协调力度，推动县域经济跨越发展。扩大经济发达镇经济社会管理权限，推动镇级市试点，做大做强乡镇经济。支持孝南发展纸制品、家具森工等特色产业，培育7大百亿产业集群〔23〕。支持汉川做大工业规模，进入全省一类县市区先进行列。支持应城盐化产业转型发展，推动厨卫家居、食品医药产业实现集群发展。支持云梦拓展“一区三园”〔24〕，唱响“楚国别都、黄香故里、古泽云梦”旅游名片。支持安陆建设全国农产品加工示范区，提升“李白故里、银杏之乡”知名度。支持大悟发展绿色能源、科教文化等特色产业，打造生态文化旅游区。支持孝昌机械电子产业进入全省重点成长型产业集群，加快建设全省山区经济强县。

　　（三）把改革创新作为适应经济新常态的根本动力。通过深化改革，增强发展的新动力；通过创新驱动，拉动发展的新引擎；通过大众创业，激发市场的新活力。

　　按照市委全面深化改革的实施意见，有力有序推进改革。在政府职能转变方面，坚持把改革贯穿政府工作始终，以政府自身革命为引领，带动主要领域改革。完成政府机构改革的后续工作；做好国务院、省政府新取消和下放的行政审批事项清理和承接工作；深化“三集中三到位”〔25〕改革，动态调整市级行政审批事项目录；完善市级行政权力清单、负面清单和责任清单；深入推进事业单位分类改革，加强综合执法、城市管理体制改革，完成公车制度改革。在经济领域，推进要素市场化配置改革，促进要素自由流动；出台加快非公有制经济健康发展、构建开放型经济新体制、创新开发园区体制机制的意见；推进国企国资改革，深化物价、财税、金融体制改革；启动不动产登记工作，加强产权市场建设，建立农村产权交易平台，全面开展农村土地承包经营权确权登记颁证。在社会领域，全面推进国家中小学教育质量综合评价改革试验区建设；开展分级诊疗、医养结合试点，抓好8家县级公立医院综合改革试点；启动机关事业单位养老保险制度改革，加强城乡养老保险制度衔接。

　　发挥科教、军工优势，大力推动创新驱动发展。加强科技与经济对接、创新成果与产业对接，以高新技术改造传统产业，以新业态、新产业推动经济转型升级。转化重大科技成果30项。发挥企业在创新中的主体地位，依托武汉及本地高等院校、科研院所，组建市级企业技术创新联盟。新建省级企业研发平台3-5家，争取国家和省科研项目50项。搭建军民融合信息交流平台，推动军工企业利用设备、技术、人才优势，寻求战略合作者，鼓励军工企业开展合资合作、扩大民品生产，支持本地企业配套生产军品。加大高新技术企业培育力度，新增高新技术企业20家，高新技术产业增加值增长12%。

　　围绕释放创造潜能，鼓励大众创业、全民创业。简化行政审批，打破市场壁垒，激发市场活力。完善创业服务平台，建立政策激励机制，组织实施“六大创业工程”〔26〕，支持有专利、有技术、有创业意愿的人创业。继续实施“槐荫人才计划”，大力集聚创新人才。深入实施回归创业工程，新创办投资500万元以上回归企业600家。

　　(四)把公共服务均等化作为推动社会事业进步的目标。努力缩小城乡之间生活水平和公共服务的差距，全力促进社会公平公正，织牢社会安全、食品药品安全、安全生产“三张网”，确保人民群众共享发展成果。

　　以彰显孝文化特色为着力点，推动文化事业迈出新步伐。用好孝文化研究成果，开展孝廉、孝德、孝亲、孝诚系列活动。关心下一代健康成长。办好中华孝文化旅游节。新建一批村级（社区）文化广场，加强农家书屋管理利用，实施全民健身工程,引导人民群众开展丰富多彩、健康向上的文体活动。做大孝感日报传媒集团、广电传媒集团等文化龙头企业。

　　以促进教育公平为方向，协调发展各类教育。新建、续建教育项目11个，对322所学校进行“全面改薄”〔27〕。实施“城乡校际联盟行动计划”，推进校际教育教学一体化改革，促进优质教育资源均衡利用。实施第二期学前教育三年行动计划，积极发展特殊教育。优化现代职业教育发展规划，启动湖北工程技师学院建设，加快职教资源整合，创新职教人才培养模式。支持湖北工程学院、湖北职业技术学院建设发展。加强民办教育培训机构监管。

　　以夯实基层基础为重点，为人民群众提供满意的卫生计生服务。推动城市卫生优质资源向农村倾斜，推进市县、县乡医院医疗联合体建设，缓解“看病难”。创新支付方式，加强价格监管，规范医疗机构服务行为，缓解“看病贵”。实施孝感中医院、孝昌第一人民医院、云梦城东医院、应城和安陆妇幼保健院等改扩建项目，改造提升8个乡镇卫生院。关心关爱计划生育特殊家庭，出生人口性别比下降2个点。

　　以最严格的全过程监管为手段，确保食品药品安全。建设农产品标准化生产示范区，支持云梦创建全国农产品质量安全县，农产品质量安全总体合格率达到96％以上。加强食品药品技术支撑体系建设，建立健全生产、加工、流通、消费环节追溯体系。对城镇餐饮行业实行信息公示制度，对农村集体聚餐实行备案管理。对城镇药品零售企业实行电子监控全覆盖，对乡村医疗机构药品强化规范管理。治理“餐桌污染”，依法严厉打击“黑工厂、黑作坊、黑窝点”，确保人民群众吃得放心、用得安心。

　　以完善体制机制为方向，推动社会治理创新。加强社会组织、专业机构建设，增强社会调适功能。推进公共服务、市场服务、志愿服务进社区，深化网格化管理，增强三个“1+X”服务体系〔28〕活力。加强村级资产、资金、资源管理和监督，推进村务公开规范化、常态化。深化城乡警务改革，构建适应动态治安特点的立体化社会治安防控体系，建设法治孝感、平安孝感。深入开展综治信访维稳“六大行动”〔29〕、健全完善“十大机制”〔30〕，全面推进“阳光信访”和依法逐级走访，支持群众理性表达诉求、依法维护权益。加强源头控制，开展隐患排查，实施专项整治，确保安全生产形势持续稳定。

　　加强国防教育、民兵预备役建设，争创全国双拥模范城“七连冠”、全省双拥模范城“八连冠”。加强人防建设，发挥好人防设施的城市功能和经济社会效益。发展慈善事业，落实民族宗教政策，做好外事侨务、统计调查、对台、老龄、残联、地方志、档案、保密等工作，支持工会、共青团、妇联、工商联、科协等群团组织依照各自章程开展活动，支持中央、省驻孝单位建设和发展。

　　（五）把民生改善作为政府工作的落脚点。更加注重保障基本民生，兜住底线，更加注重困难群体、特殊人群的就业救助，更加注重生态环境的保护和改善，努力让人民群众过上有质量的生活。

　　坚持把就业作为最大的民生，最大限度地实现比较充分的就业。深入推进“双助”行动，实施大学毕业生就业促进计划，突出做好重点人群的就业工作。引导转变就业观念，发展与就业政策相适应的职业教育培训，推进就业机会平等，让更多的人都能通过自己的努力，掌握与市场需求相适应的就业技能、获得应有的岗位。

　　坚持保障和救助“两手抓”，构建更加牢靠便捷的社保“安全网”。推进城乡一体化的社会养老、医疗保险体系建设，完善被征地农民社会养老保险机制。拓展社会保障卡“一卡通”应用功能，方便人民群众就医。完善社会救助、社会福利、灾害救助应急体系，加快城市社会福利中心建设，支持社会力量举办养老机构，扩大城市居家养老服务覆盖范围。加大保障性住房建设、分配和管理力度，新开工保障性住房5.9万套，建成1.9万套。规范小区物业管理，扩大住房公积金制度覆盖面。

　　坚持保护、建设、治理“三位一体”，建设生态孝感。策划建设一批生态、环保项目，推进武汉城市圈“两型”社会试验区建设，支持大别山试验区突出发展生态产业。推进“绿满澴川”行动，支持孝昌、安陆创建省级林业生态示范县，支持孝南朱湖、汉川汈汊湖、安陆府河国家湿地公园建设，加强老灌湖、云梦曲阳湖等湿地保护。推进城市公园、街头绿地建设，确保国家园林城市创建验收达标。加大闲置土地清理处置力度，深化国土资源节约集约模范县市创建工作。加强重点湖泊生态治理修复，严格落实人大决议，保护好王母湖、野猪湖。实施槐荫河、府澴河流域水污染综合治理。推广清洁燃料，严控交通和施工扬尘，实施机动车环保标志管理，确保空气质量不断改善。推进农村生活垃圾治理五年行动，整治农业面源污染。健全完善一企一档、重点污染源在线监控制度，确保达标排放。两年内全部关闭双峰山沿线采石场。

　　各位代表，市政府今年将继续办好“十件实事”。①实施困难群众临时救助制度；新增城镇就业4.5万人以上，“双助”帮扶2000名困难人员就业创业。②建立健全贫困学生资助体系，资助各类贫困学生6万人以上，资助资金1.2亿元以上。③医疗救助贫困群众2.6万人以上；为孝感城区60岁以上老年人购买意外伤害综合保险；帮助解决残疾人生活困难。④为留守老人、留守儿童建立数据库和健康档案，免费提供健康服务。⑤推进基层食品药品监管机构全覆盖，保障人民群众饮食用药安全。⑥实现农村客运“村村通”；孝感城区新增公交线路2条。⑦改造孝感城区20条背街小巷，新建改造60条街巷路灯，优化改造5处路口。⑧实施槐荫河截污工程，改善槐荫河水质。⑨推进文昌中学、丹阳中学、实验小学、玉泉小学与市直“三区”4所学校开展合作办学试点，实现教育教学一体化管理。⑩免费定向委培100名新型乡村医生。

　　三、以法治政府建设为核心，加强政府自身建设

　　巩固党的群众路线教育实践活动成果，认真开展“三严三实”专题教育活动。坚持依法行政、执政为民，切实做到对人民负责、受人民监督、让人民满意。

　　坚持做到依法依规、全面履行职能。编制实施《孝感市加强法治政府建设规划》，在政府部门普遍建立法律顾问制度，用法治引领和规范政府行为。加大向社会力量购买政府服务力度，用法治化、市场化、社会化的手段履行好政府职能。开展法治单位创建活动，建立健全基层法律服务体系，为困难群众提供法律援助，圆满通过“六五”普法验收。建立领导干部学法用法制度，提升用法治思维和法治方式解决实际问题的能力。落实公务员履职问责规定 ,重点对慢作为、不作为、乱作为行为进行追究。

　　坚持做到严格规范、公正文明执法。把执法管理力量向基层一线倾斜，重点加大对食品药品、工商质检、公共卫生、安全生产、环境保护等领域的执法力度。完善执法程序，建立执法全过程记录制度，从严惩处执法违法行为。提倡言行文明，热情服务，实现行政执法的公正化、人性化。畅通举报投诉渠道，让人民群众的诉求得到及时有效回应。

　　坚持做到主动接受监督、阳光运行权力。强化权力监督制约，做到依法用权、秉公用权、廉洁用权。加强对行政审批、项目招投标、土地出让、政府采购、国有资产产权交易等重点领域的监管。健全完善政府重大决策和重要事项事前向人大报告、与政协协商机制，采取现场督办、当面回复等方式，提高人大议案建议和政协提案办理实效。广泛听取民主党派、工商联、无党派人士和人民团体意见建议，高度重视司法监督、舆论监督和社会公众监督。开展有立法权城市的申报工作。加快电子政务平台建设，加大政府信息公开力度，让权力公开透明运行。完善对同类项目整体打包招标建设机制，全方位加强重大工程监督。

　　坚持做到务实高效、清正廉洁。坚决落实中央八项规定精神、省委六条意见和市委七十五条要求，严格执行“四风”整治〔31〕的各项制度，进一步精简会议和文件，推动政府系统作风建设常态化、长效化。坚持“三情”结合破解发展难题，实实在在谋事创业。强化责任担当，每名市政府班子成员、每个政府部门确定3—5个重点难点工作，定期督查推进落实情况。改进抓落实的方式方法，做到发现问题在一线、解决问题在一线、检查督办在一线、落实到位在一线。认真落实党风廉政建设责任制，落实市政府领导、政府部门负责人主体责任，严以修身、严以律已，做到心中有党、心中有民、心中有责、心中有戒。管好班子、带好队伍，形成实实在在做人、干干净净做事的清廉政风。进一步加大惩治和预防腐败工作力度，支持纪检监察机关严肃查处违纪违法案件，坚决纠正损害群众利益的不正之风。

　　各位代表，让我们在市委的坚强领导下，勇担重任，励精图治，锐意进取，努力完成“十二五”目标任务，大力推进“五个跨越”、加快建设“五个城市”，谱写孝感人民美好生活的新篇章！
